# Effect of Black Tea Consumption on Urinary Risk Factors for Kidney Stone Formation

**DOI:** 10.3390/nu13124434

**Published:** 2021-12-11

**Authors:** Roswitha Siener, Albrecht Hesse

**Affiliations:** Department of Urology, University Stone Center, University Hospital Bonn, 53127 Bonn, Germany; albrecht-hesse@web.de

**Keywords:** tea, black tea, caffeine, oxalate, hyperoxaluria, urinary stone formation, urolithiasis, kidney stone, nephrolithiasis

## Abstract

Copious fluid intake is the most essential nutritional measure in the treatment of urolithiasis, and is suggested to be a protective factor in the primary prevention of urinary stone formation. Although the intake of black tea contributes to daily fluid intake, the high oxalate content could outweigh the beneficial effect of urine dilution. The present study investigated the effect of black tea consumption on urinary risk factors for kidney stone formation. Ten healthy men received a standardized diet for a period of ten days. Subjects consumed 1.5 L/day of fruit tea (0 mg/day oxalate) during the 5-day control phase, which was replaced by 1.5 L/day of black tea (86 mg/day oxalate) during the 5-day test phase. Fractional and 24-h urines were obtained. The intake of black tea did not significantly alter 24-h urinary oxalate excretion. Urinary citrate, an important inhibitor of calcium stone formation, increased significantly, while the relative supersaturation of calcium oxalate, uric acid, and struvite remained unchanged. No significantly increased risk for kidney stone formation could be derived from the ingestion of black tea in normal subjects. Further research is needed to evaluate the impact of black tea consumption in kidney stone patients with intestinal hyperabsorption of oxalate.

## 1. Introduction

Urinary stone disease is a significant economic burden on healthcare systems, which is likely to increase with time [1,2,3]. The prevalence and incidence of urolithiasis has increased globally over the last several decades [4]. The prevalence of urinary stones was reported to be 5% in Germany and 10% in the United States [5,6]. The stone recurrence rate, which was estimated to be around 50% after 10 years, is alarmingly high [7,8]. Low urine volume is a major risk factor for kidney stone formation [9]. Copious fluid intake is the most essential nutritional measure in the treatment of urolithiasis, regardless of stone type and specific risk factors for stone formation [9,10,11]. Moreover, increased fluid intake was found to be associated with a reduced risk of incident nephrolithiasis in both women and men [12,13,14,15,16].

Tea is among the most widely consumed beverages worldwide, and contributes to daily fluid intake. Several systematic reviews assumed a potentially protective effect of tea against urinary stone formation [17,18,19]. The beneficial effects of tea were attributed primarily to the diuretic action from the intake of substantial amounts of caffeine [17,18]. Further explanations for the preventive role of tea consumption in these studies were the antioxidant capacity related to the polyphenol content of tea, and the addition to the daily fluid intake [18,19].

A limitation of these epidemiological studies is that no distinction was made between different tea types, particularly black and green tea, which both originate from *Camellia sinensis*, and herbal tea. Moreover, tea consumption patterns that may affect intestinal oxalate absorption were not considered—that is, if regular tea was consumed with calcium-rich meals or contained added milk. In general, different teas contain varying concentrations of oxalate. While the oxalate content of herbal and fruit tea was reported to be low, green and black tea were found to have the highest amounts of oxalate [20,21,22]. Increased urinary oxalate excretion following high dietary ingestion and/or hyperabsorption of oxalate is a major lithogenic risk factor. It is suggested that dietary oxalate considerably contributes to urinary oxalate excretion, even in healthy subjects without oxalate hyperabsorption [23,24]. Black tea, a popular beverage and an important source of dietary oxalate, could be a relevant dietary risk factor for calcium oxalate stone formation.

However, previous studies that addressed the effect of the ingestion of brewed black tea without milk on urinary oxalate excretion in healthy subjects have yielded inconsistent results. While Finch et al. [25] reported a rise in urinary oxalate excretion of 0.130 mmol/24 h after the consumption of 1.0 L of tea containing 48–55 mg oxalate, Brinkley et al. [26] found an increase in urinary oxalate excretion of only 0.013 mmol over an 8-h period after ingestion of 0.5 L of brewed black tea without milk. Apart from urinary oxalate and calcium excretion, data on the impact of black tea ingestion on further urinary promoters and inhibitors of lithogenesis are lacking. Therefore, the present study investigated the effect of brewed black tea consumption on the urinary risk profile for kidney stone formation in normal subjects under controlled standardized conditions.

## 2. Materials and Methods

### 2.1. Study Subjects

Ten healthy men were recruited for the study. Only men were included in order to ensure a homogenous group. Study exclusion criteria comprised a history of kidney stone formation or any other disease. Each study participant had a normal medical examination, normal findings from urine dipstick (Combur 9 test; Roche Diagnostics GmbH, Mannheim, Germany), and two 24-h urine analyses prior to the start of the study. Subjects took no medications or dietary supplements during the study. The study was approved by the Ethics Committee of the Medical Faculty of the University of Bonn (01790), and informed consent was obtained.

### 2.2. Study Procedure

Participants received a standardized diet during the whole study period of ten days. The standardized diet was calculated using PRODI 5.3 (Nutri-Science, Freiburg, Germany). The standardized diet corresponded to a balanced mixed diet and provided a constant total fluid intake with beverages of 2.5 L/day. After a few days of adaptation, the standardized dietary regimen (i.e., the constant daily intake of prescribed foods and fluids) leads to a steady state of metabolism so that constant urinary values are achieved [27].

The study lasted ten days and comprised two phases, a 5-day control phase and a 5-day test phase. During the test phase, 1.5 L/day of fruit tea (0 mg/day oxalate) was replaced by 1.5 L/day of brewed black tea (86 mg/day oxalate). The fruit tea was prepared by steeping 6 tea bags, each containing 3 g, corresponding to a total of 18 g of tea, in 1.5 L 70 °C water for 5 min. For black tea, 12 tea bags, each containing 1.75 g, corresponding to a total of 21 g tea leaves, were infused in 1.5 L 70 °C water for 5 min. Except for oxalate (0 mg/L vs. 57.5 mg/L), the concentrations of sodium (23 mg/L vs. 23 mg/L), potassium (117 mg/L vs. 156 mg/L), calcium (75 mg/L vs. 39 mg/L), and magnesium (28 mg/L vs. 22 mg/L) were similar in fruit tea and black tea.

During the study period, participants collected daily 24-h urine. On day 5 of the control and test phases, fractional urine was collected to determine the circadian excretion of urinary parameters. Subjects were instructed to drink 300 mL of the fruit tea (control phase) and the black tea (test phase) at fixed times (7.00, 10.00, 13.00, 16.00, and 19.00 h). Urine was collected at five 3-h intervals during the daytime (7.00–22.00 h) and in one 9-h interval during the night (22.00–7.00 h). All parameters were determined in each urine fraction and the resulting 24-h urine.

### 2.3. Urinary Parameters

Urine volume, density, pH (potentiometry), urinary sodium and potassium (flame emission spectrophotometry), magnesium and calcium (atomic absorption spectroscopy), ammonium (ion selective electrode), chloride (coulomb metric titration), inorganic sulfate (nephelometry), inorganic phosphate (phosphate molybdate reaction), creatinine (Jaffé reaction), uric acid (enzymatically, uricase), citrate (enzymatically, citrate lyase), and oxalate (ion chromatography) were analyzed [28]. Laboratory quality certification was available for each parameter. The risk of stone formation, computed as relative supersaturation of calcium oxalate, uric acid, and struvite, was calculated using the computer program EQUIL2 (University of Florida, Gainesville, FL, USA) [29].

### 2.4. Statistical Analysis

A statistical comparison of differences between the urinary parameters of the control phase and the test phase within the study group was performed using the nonparametric Wilcoxon matched-pairs signed-rank test. Data are presented as mean ± standard deviation (SD). The last day of each phase served as control and test days, respectively, since steady-state conditions were then attained. The significance level was considered as *p* < 0.05. All statistical tests were two-sided. Statistical analysis was performed using SPSS^®^ for Windows version 27.0 (IBM, Armonk, New York, NY, USA).

## 3. Results

Ten men with a mean age of 26.3 ± 3.3 years (range: 20–31 years) were included in the study. The mean body weight, height, and body mass index of the study participants were 80.0 ± 11.1 kg, 178.7 ± 5.1 cm, and 25.1 ± 3.3 kg/m^2^, respectively.

The 24-h urine composition on the last day of the control and test phases is presented in Table 1. Urinary excretion of citrate increased, while urinary oxalate excretion and the relative supersaturation of calcium oxalate, uric acid, and struvite remained unchanged after the intake of black tea. No change in any other 24-h urinary parameter was observed following black tea consumption.

**Figure 1:**
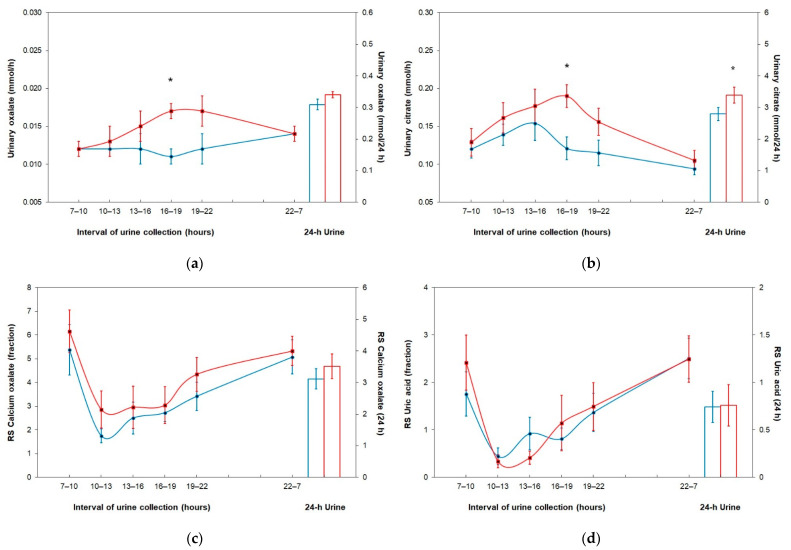
Fractional and 24-h urine parameters after the consumption of fruit tea (control day, blue line) and black tea (test day, red line); data are represented as M ± SEM. (**a**) Urinary oxalate excretion; (**b**) urinary citrate excretion; (**c**) relative supersaturation of calcium oxalate; (**d**) relative supersaturation of uric acid. Abbreviation: RS, relative supersaturation. * Wilcoxon test within groups: <0.05.

Figure 1 compares fractional and 24-h urine oxalate excretion, citrate excretion, relative supersaturation of calcium oxalate, and relative supersaturation of uric acid on the control day and the test day. Except for a significantly higher excretion in the fourth urine fraction, no difference in urinary oxalate excretion was observed in any other urine fraction or 24-h urine on the black tea loading (Figure 1a). Urinary citrate excretion was significantly higher in the fourth urine fraction and in 24-h urine during black tea consumption (Figure 1b). No significant change in the relative supersaturation of calcium oxalate (Figure 1c) and uric acid (Figure 1d) occurred, neither in any urine fraction nor in 24-h urine, after the ingestion of black tea.

The circadian course of urinary parameters is presented in Table 2. After the intake of black tea, urine volume and urinary excretion of sodium, chloride, calcium, magnesium, uric acid, oxalate, and citrate were significantly higher in the fourth urine fraction on the test day compared to the control day. No change in the relative supersaturation of calcium oxalate, uric acid, and struvite occurred in any urine fraction during the consumption of black tea.

## 4. Discussion

Black tea, prepared from the leaves of *Camellia sinensis*, is a rich source of polyphenols, particularly theaflavins and thearubigins, which exhibit antioxidant activity in vitro and in vivo [30,31]. Tea is increasingly being studied for its beneficial effects against several chronic diseases, including cardiovascular diseases and cancer [30,31]. Furthermore, several systematic reviews have suggested a protective role of tea intake on the development of kidney stones [17,18,19]. Despite the potentially preventive effect of black tea on urinary stone formation, a high content of the anti-nutrient oxalate could negate the favorable effect of urine dilution.

The oxalate content of black tea that was examined in the present study amounted to 57.5 mg/L oxalate, which is within the range of oxalate concentrations previously reported for black tea [21,22,32,33]. A total of 1.5 L/day of black tea containing 86 mg/day soluble oxalate was administered to evaluate the effect of a high tea load on urinary risk factors for kidney stone formation, particularly on urinary oxalate excretion and the risk of calcium oxalate stone formation. Following the intake of 1.5 L/day of black tea, urinary oxalate excretion increased by 0.031 mmol/day on average, although not significantly. Assuming that oxalate from a specific foodstuff is not significantly metabolized after absorption, its excretion after the intake of a known amount estimates bioavailability [34]. Using this approach, the bioavailability of oxalate from black tea amounted to only 3.2%.

Previous studies that assessed the bioavailability of oxalate from black tea without milk in normal subjects have yielded a wide range. The oxalate bioavailability was reported to be only 0.08% over an 8-h period after the consumption of 500 mL of brewed black tea [26], whereas oxalate absorption from two brands of black tea (60.4 and 62.4 mg oxalate) ranged from 1.9 to 4.7% over a 6-h urine collection period [35]. Liebman et al. [36] observed a mean oxalate absorption rate of 2.4% during a 6-h period after the administration of 25 mg of ^13^C_2_-oxalate provided in conjunction with the ingestion of 600 mL of black tea containing 40 mg oxalate. However, it should be noted that oxalate bioavailability in these studies is an underestimate of true absorption because additional oxalate would have been recovered if the urine collection period had been extended to 24 h. The research group yielded an extrapolated estimate of 2.9% absorption of oxalate if urine had been collected over a 24-h period [36]. Therefore, the results of the present study are in accordance with the previously reported bioavailability of oxalate from brewed black tea [35,36]. In contrast, an earlier study found a 22% absorption rate of oxalate over a 24-h urine collection period after the consumption of black tea [25]. However, due to the small sample size of only three participants and the substantial intersubject variability, the validity of this observation is limited.

As opposed to healthy subjects, intestinal oxalate absorption was reported to be significantly higher in urinary stone patients [37]. Using the standardized ^13^C_2_-oxalate absorption test, oxalate absorption greater than 10% was found in 46% of idiopathic calcium oxalate stone formers as opposed to 28% of healthy subjects [37]. Considering this finding, there is overall support for the recommendation that calcium oxalate stone patients, particularly those with intestinal hyperabsorption of oxalate, limit their consumption of black tea. In a recent case–control study, a high consumption of tea was associated with an increased risk of calcium oxalate stone formation [38]. In contrast to black tea, the fruit tea used as a control in the present study is derived from completely different plants (i.e., a mixture of berries, dried fruits, leaves, flowers, and citrus peels). Due to the composition of the fruit tea, the oxalate content was below the detection limit. In general, herbal and fruit teas have been recommended as an alternative to teas from *Camellia sinensis* as they have much lower oxalate concentrations [11,21,22,34].

Although it is assumed that soluble oxalate in black tea binds to calcium in the intestinal tract, thereby reducing calcium absorption and excretion, no change in 24-h urinary calcium excretion was observed after the consumption of black tea in the present study. Therefore, it is reasonable to suggest that the absorption of calcium was not affected by the oxalate content of black tea, which could be explained by the timing of tea ingestion. The study participants were instructed to consume the tea between meals in order to avoid any interaction between components of the tea and their diet. Because intestinal oxalate absorption depends strongly on dietary calcium intake [39], it is suggested that the ingestion of black tea with milk or with calcium-containing meals could reduce the highly bioavailable soluble oxalate for absorption. A previous study in healthy subjects found that urinary oxalate excretion was significantly lower when black tea was consumed with milk [35].

Interestingly, the consumption of 1.5 L/day of black tea resulted in a significant increase in urinary citrate excretion by 21% over the 24-h urine collection period. Urinary citrate is an important inhibitor of calcium stone formation. Citrate inhibits stone formation by complexing with calcium in the urine, inhibiting spontaneous nucleation, and preventing the growth and agglomeration of crystals [40]. Urinary citrate excretion is mainly influenced by the rate of citrate absorption from the glomerular filtrate and metabolism by the proximal tubule cell [41]. Changes in acid–base homeostasis are the predominant physiological determinant of proximal tubule reabsorption and urinary excretion of citrate [42,43]. In the present study, urinary citrate excretion increased with the ingestion of black tea, apparently in the absence of a change in urinary pH or potassium excretion. One hypothesis could be that other modulators of citrate metabolism (e.g., organic anions) may have caused an increase in citrate excretion in the urine by competing at the transporter [42,43]. However, studies on the effect of major organic acids of black tea infusions, other than oxalic acid (e.g., citric, malic, quinic, or succinic acid) are lacking.

Alongside coffee, tea is also a prominent source of caffeine in the diet. Caffeine intake has been reported to be associated with a lower risk of incident kidney stones [44]. The caffeine content of black tea infusion was reported to be approximately 220 mg/L in [45]. The European Food Safety Authority considers the habitual caffeine consumption of up to 400 mg/day an amount at which non-pregnant healthy adults are not at risk of potential adverse effects [46]. The diuretic effect of caffeine is well established, and can be explained by an interaction with the adenosine receptor A_1_ in the renal proximal tubule, leading to inhibition of renal reabsorption and causing diuresis and natriuresis [46,47]. Moreover, caffeine has been reported to increase the urinary excretion of calcium, magnesium, potassium, and chloride [48]. In the present study, the significantly higher urine volume, sodium, chloride, calcium, and magnesium excretion in the fourth urine fraction on the test day could be attributed to the cumulated intake of substantial quantities of caffeine. Because the diuretic action of caffeine and the elevated urinary citrate excretion offset the increased calcium excretion, the relative supersaturation of calcium oxalate remained unchanged in the fourth urine fraction after the ingestion of black tea. Moreover, the caffeine intake with black tea did not lead to significant changes in 24-h urine volume, specific gravity, or mineral excretion compared to the control. Further research is required to clarify the role of caffeine intake in urinary uric acid excretion.

The comprehensive analysis of all relevant urinary risk factors for stone formation enabled the assessment of the effect of black tea consumption on the risk of forming specific types of stones, computed as the relative supersaturation of the stone-forming components. No change in the risk of stone formation for calcium oxalate, uric acid, or struvite was observed, neither in any urine fraction nor in 24-h urine. Although urinary oxalate, calcium, and uric acid excretion were significantly higher in the fourth fraction of urine collection after the ingestion of black tea compared to the control phase, the relative supersaturation of calcium oxalate and uric acid did not change due to the significantly higher urine volume and citrate excretion.

A limitation of our study is the relatively small number of participants. As the study was conducted under strictly controlled conditions, a steady state of metabolism can be assumed [27]. Further research is required to evaluate the impact of habitual black tea consumption on the risk of stone formation, particularly in calcium oxalate stone patients with intestinal hyperabsorption of oxalate.

## 5. Conclusions

To our knowledge, this is the first study to evaluate the impact of black tea consumption on all relevant urinary promoters and inhibitors of lithogenesis, in addition to urinary calcium and oxalate excretion. Interestingly, urinary citrate excretion—an important inhibitor of calcium stone formation—increased significantly after the ingestion of black tea. In contrast, the consumption of the oxalate-rich black tea did not significantly alter 24-h urinary oxalate excretion. Urine volume, sodium, chloride, calcium, and magnesium excretion were significantly higher in the fourth urine fraction after the ingestion of black tea, which could be attributed to the cumulated intake of substantial quantities of caffeine. However, the diuretic action of caffeine intake with black tea did not lead to significant changes in 24-h urine volume, specific gravity, or mineral excretion compared to the control. No significantly increased risk of kidney stone formation could be derived from the ingestion of black tea in normal subjects. Further research is needed to evaluate the impact of habitual black tea consumption in kidney stone formers with intestinal hyperabsorption of oxalate.

## Figures and Tables

**Table 1:** Twenty-four-hour urine composition after the consumption of fruit tea (control day) and black tea (test day) (*n* = 10).

	Fruit Tea	Black Tea	*p*-Value
	Mean ± SD	Mean ± SD	
Volume (L/24 h)	2.477 ± 0.504	2.378 ± 0.420	0.684
Density (g/cm^3^)	1.008 ± 0.002	1.008 ± 0.001	0.813
pH	6.27 ± 0.29	6.28 ± 0.35	0.959
Sodium (mmol/24 h)	205 ± 47	206 ± 22	0.959
Potassium (mmol/24 h)	72 ± 18	68 ± 14	0.386
Calcium (mmol/24 h)	5.41 ± 1.81	5.40 ± 1.56	0.879
Magnesium (mmol/24 h)	5.11 ± 1.13	5.16 ± 0.86	0.647
Ammonium (mmol/24 h)	36.8 ± 6.6	38.6 ± 11.0	0.575
Chloride (mmol/24 h)	205 ± 49	204 ± 21	0.959
Phosphate (mmol/24 h)	31.1 ± 3.5	31.6 ± 5.1	0.799
Sulfate (mmol/24 h)	20.4 ± 4.4	17.6 ± 4.3	0.169
Creatinine (mmol/24 h)	18.20 ± 2.02	17.32 ± 1.58	0.203
Uric acid (mmol/24 h)	3.58 ± 0.53	3.40 ± 0.43	0.169
Oxalate (mmol/24 h)	0.309 ± 0.052	0.340 ± 0.032	0.139
Citrate (mmol/24 h)	2.793 ± 0.664	3.387 ± 0.789	0.002
RS Calcium oxalate	3.117 ± 1.032	3.514 ± 1.249	0.386
RS Uric acid	0.741 ± 0.519	0.760 ± 0.692	0.799
RS Struvite	0.065 ± 0.069	0.082 ± 0.083	0.285

Abbreviations: RS, relative supersaturation; SD, standard deviation.

**Table 2:** Fractional urine composition after the consumption of fruit tea (control day) and black tea (test day) (*n* = 10).

	Fruit Tea						Black Tea					
	Interval of Urine Collection (Hours)						Interval of Urine Collection (Hours)					
	7–10	10–13	13–16	16–19	19–22	22–7	7–10	10–13	13–16	16–19	19–22	22–7
	Mean ± SD	Mean ± SD	Mean ± SD	Mean ± SD	Mean ± SD	Mean ± SD	Mean ± SD	Mean ± SD	Mean ± SD	Mean ± SD	Mean ± SD	Mean ± SD
Volume (L/h)	0.080 ± 0.041	0.219 ± 0.108	0.154 ± 0.082	0.113 ± 0.041	0.085 ± 0.036	0.058 ± 0.025	0.064 ± 0.026	0.158 ± 0.079 *	0.145 ± 0.067	0.172 ± 0.056 *	0.107 ± 0.046	0.049 ± 0.016
Density (g/cm^3^)	1.012 ± 0.005	1.005 ± 0.002	1.008 ± 0.005	1.007 ± 0.005	1.011 ± 0.006	1.015 ± 0.005	1.013 ± 0.006	1.006 ± 0.004	1.008 ± 0.004	1.007 ± 0.004	1.010 ± 0.004	1.014 ± 0.004
pH	6.03 ± 0.47	6.47 ± 0.53	6.46 ± 0.58	6.20 ± 0.40	6.08 ± 0.35	5.83 ± 0.32	5.84 ± 0.25	6.63 ± 0.46	6.59 ± 0.36	6.33 ± 0.70	5.98 ± 0.58	5.83 ± 0.41
Sodium (mmol/h)	7.75 ± 2.73	12.51 ± 7.82	12.72 ± 6.10	7.74 ± 2.76	8.68 ± 3.98	6.34 ± 1.60	6.49 ± 3.76	11.17 ± 3.75	11.88 ± 4.70	12.08 ± 3.64 *	10.63 ± 6.47	5.48 ± 1.50
Potassium (mmol/h)	3.08 ± 1.39	4.37 ± 1.96	4.87 ±1.49	2.45 ± 1.12	2.88 ± 1.52	2.12 ± 0.64	2.99 ± 1.51	4.91 ± 1.89	4.41 ± 2.45	2.83 ± 0.80	2.51 ± 1.18	1.65 ± 0.49 *
Calcium (mmol/h)	0.270 ± 0.120	0.377 ± 0.241	0.290 ± 0.139	0.243 ± 0.118	0.217 ± 0.134	0.135 ± 0.053	0.259 ± 0.145	0.286 ± 0.121	0.271 ± 0.124	0.333 ± 0.122 *	0.270 ± 0.122	0.127 ± 0.048
Magnesium (mmol/h)	0.228 ± 0.073	0.253 ± 0.121	0.212 ± 0.056	0.213 ± 0.081	0.218 ± 0.115	0.194 ± 0.053	0.224 ± 0.073	0.195 ± 0.06	0.208 ± 0.09	0.301 ± 0.09 *	0.249 ± 0.09	0.181 ± 0.068
Ammonium (mmol/h)	1.78 ± 0.53	1.81 ± 0.53	1.29 ± 0.52	1.49 ± 0.54	1.30 ± 0.46	1.54 ± 0.43	1.80 ± 0.60	1.39 ± 0.71	1.39 ± 0.86	2.07 ± 1.02	1.76 ± 0.41 *	1.48 ± 0.66
Chloride (mmol/h)	8.97 ± 2.69	14.40 ± 7.83	13.04 ± 5.53	7.54 ± 2.42	7.87 ± 3.79	5.51 ± 1.51	8.29 ± 4.24	13.55 ± 3.97	12.18 ± 5.20	10.82 ± 3.43 *	9.53 ± 5.37	4.49 ± 1.33
Phosphate (mmol/h)	0.87 ± 0.44	0.89 ± 0.38	1.30 ± 0.53	1.28 ± 0.33	1.63 ± 0.53	1.47 ± 0.21	0.81 ± 0.34	0.67 ± 0.39	1.32 ± 0.50	1.75 ± 0.82	1.79 ± 0.61	1.39 ± 0.41
Sulfate (mmol/h)	0.77 ± 0.33	0.75 ± 0.35	0.85 ± 0.27	0.89 ± 0.33	0.97 ± 0.44	0.86 ± 0.15	0.71 ± 0.37	0.63 ± 0.24	0.75 ± 0.33	0.95 ± 0.38	0.83 ± 0.40	0.66 ± 0.21 *
Creatinine (mmol/h)	0.759 ± 0.199	0.674 ± 0.163	0.752 ± 0.172	0.718 ± 0.211	0.716 ± 0.278	0.815 ± 0.122	0.801 ± 0.278	0.691 ± 0.296	0.669 ± 0.274	0.840 ± 0.239	0.751 ± 0.191	0.673 ± 0.210 *
Uric acid (mmol/h)	0.155 ± 0.066	0.181 ± 0.063	0.177 ± 0.047	0.140 ± 0.039	0.133 ± 0.054	0.136 ± 0.031	0.144 ± 0.058	0.165 ± 0.058	0.163 ± 0.062	0.194 ± 0.064 *	0.128 ± 0.034	0.113 ± 0.038 *
Oxalate (mmol/h)	0.012 ± 0.004	0.012 ± 0.005	0.012 ± 0.005	0.011 ± 0.003	0.012 ± 0.005	0.014 ± 0.004	0.012 ± 0.003	0.013 ± 0.005	0.015 ± 0.006	0.017 ± 0.004 *	0.017 ± 0.005	0.014 ± 0.003
Citrate (mmol/h)	0.012 ± 0.038	0.139 ± 0.044	0.154 ± 0.073	0.121 ± 0.048	0.115 ± 0.052	0.094 ± 0.026	0.129 ± 0.058	0.161 ± 0.065	0.177 ± 0.071	0.190 ± 0.046 *	0.156 ± 0.056	0.105 ± 0.042
RS Calcium oxalate	5.378 ± 3.340	1.747 ± 0.946	2.504 ± 2.123	2.724 ± 1.165	3.419 ± 1.878	5.079 ± 2.243	6.151 ± 2.842	2.860 ± 2.471	2.956 ± 2.802	3.044 ± 2.467	4.345 ± 2.253	5.332 ± 1.946
RS Uric acid	1.757 ± 1.477	0.441 ± 0.549	0.923 ± 1.095	0.813 ± 0.710	1.368 ± 1.276	2.509 ± 1.339	2.419 ± 1.840	0.337 ± 0.433	0.412 ± 0.448	1.144 ± 1.851	1.493 ± 1.595	2.491 ± 1.543
RS Struvite	0.064 ± 0.070	0.026 ± 0.025	0.042 ± 0.038	0.095 ± 0.186	0.066 ± 0.088	0.073 ± 0.068	0.048 ± 0.059	0.051 ± 0.053	0.062 ± 0.061	0.056 ± 0.051	0.061 ± 0.090	0.122 ± 0.221

Abbreviations: RS, relative supersaturation; SD, standard deviation. * Wilcoxon test within groups: <0.05.

## Data Availability

Data are available on request.
